# STING palmitoylation as a therapeutic target

**DOI:** 10.1038/s41423-019-0205-5

**Published:** 2019-02-22

**Authors:** Anne Louise Hansen, Kojiro Mukai, Francisco J. Schopfer, Tomohiko Taguchi, Christian K. Holm

**Affiliations:** 1 Department of Biomedicine, Aarhus University, 8000 Aarhus C, Denmark; 2 Laboratory of Organelle Pathophysiology, Department of Integrative Life Sciences, Graduate School of Life Sciences, Tohoku University, Sendai, 980-8578 Miyagi Japan; 3 Department of Pharmacology and Chemical Biology, University of Pittsburgh, Pittsburgh, PA 15213 USA

**Keywords:** STING, Palmitoylation, Inflammation, SAVI, Interferonopathies, Inflammatory diseases, Chronic inflammation, Pattern recognition receptors, Inflammatory diseases, Chronic inflammation

## Abstract

Gain-of-function mutations in the STING-encoding gene *TMEM173* are central to the pathology of the autoinflammatory disorder *STING-*associated vasculopathy with onset in infancy (SAVI). Furthermore, excessive activity of the STING signaling pathway is associated with autoinflammatory diseases, including systemic lupus erythematosus and Aicardi–Goutières syndrome (AGS). Two independent studies recently identified pharmacological inhibitors of STING. Strikingly, both types of compounds are reactive nitro-containing electrophiles that target STING palmitoylation, a posttranslational modification necessary for STING signaling. As a consequence, the activation of downstream signaling molecules and the induction of type I interferons were inhibited. The compounds were effective at ameliorating inflammation in a mouse model of AGS and in blocking the production of type I interferons in primary fibroblasts from SAVI patients. This mini-review focuses on the roles of palmitoylation in STING activation and signaling and as a pharmaceutical target for drug development.

## Introduction

The intracellular molecule STING (Stimulator of interferon genes, also known as MPYS, ERIS, MITA, and TMEM173) is indispensable for the induction of type I interferons (IFNs, e.g., IFNα/β) in response to infection with DNA-based viruses^1–3^ and with bacteria such as *Listeria monocytogenes*,^4^ as demonstrated using both in vitro and in vivo experimental approaches. In these cases, STING acts as a sensor of cyclic dinucleotides (CDNs) that are either released into the cytosol by the bacterial pathogens^4–8^ or synthesized by the cytosolic DNA sensor cyclic GMP-AMP synthase (cGAS).^9–13^ Furthermore, STING has been demonstrated to be necessary for the optimal induction of type I IFNs by enveloped viruses through the sensing of virus-cell fusion and by RNA-based viruses through mechanisms that have not been fully elucidated.^1,14–17^

Although most reports on the function of STING center around responses to infection, the strongest links between the STING-dependent impacts on immunity and human disease have originated from the study of chronic inflammation. Inherited loss-of-function mutations in genes encoding cytosolic nucleases are strongly correlated with the development of systemic inflammatory conditions. One of the best-studied examples is the association of loss-of-function mutations in the gene *TREX1* with autoimmune diseases, including Aicardi–Goutières syndrome (ASG)^18–20^ and systemic lupus erythematosus.^21,22^ *TREX1* encodes the enzyme 3′ repair exonuclease 1 (TREX1), a 3′-5′ DNA exonuclease, which degrades cytosolic dsDNA and ssDNA.^23–25^ It has been proven difficult to directly assess the concentration of cytosolic DNA in both wild-type and TREX1-deficient cells. However, it is assumed that the loss of TREX1 activity leads to increased levels of cytosolic DNA, which then triggers the cGAS-STING pathway to release pro-inflammatory cytokines, including type I IFNs. This assumption is supported by the increased expression of type I IFNs and IFN-stimulated genes (ISGs) in both SLE^26,27^ and AGS^28^ patients. This is further supported by animal experiments, in which *Trex1*-deficient mice developed an autoimmune-like disease dependent on STING-induced type I IFN,^29,30^ which could be rescued by the knockdown of cGAS.^19,20^ Furthermore, cGAS knockdown in *Trex1*-deficient murine cells appears to rescue the increased ISG expression profile.^31^

The role of STING as a direct driver of systemic inflammation was confirmed in 2014, when both de novo^32^ and inherited^21^ gain-of-function mutations in the STING-encoding gene *TMEM173* were reported. These mutations cause STING hyperactivation, resulting in a persistent “IFN signature”. The initial finding of de novo mutations in *TMEM173*, leading to the variants V147 L, N154S, V155M, was made in six patients. The mutations cause devastating inflammatory conditions in the patients, and the disease was named *STING-*associated vasculopathy with onset in infancy (SAVI). The list of clinical symptoms can be grouped into symptoms of systemic inflammation, symptoms of peripheral vascular and skin inflammation, and pulmonary manifestations.^32^ The patients exhibit hyperactivation of STING, resulting in elevated expression of IFNβ, constitutive phosphorylation of STAT1 and a strong transcriptional ISG signature in addition to elevated levels of interferon-induced cytokines.^32^ Since then, several additional SAVI patients have been identified with both the initially described SAVI STING variants^33,34^ and recently identified variants including S102P-F279L^35^ V147M,^36^ C206Y,^37^ R281Q,^37^ R284G^37^ and R284S.^38,39^

To better investigate STING hyperactivation in vivo and to model SAVI disease, the variants N154S and V155M were introduced into mouse models using knock-in of the murine orthologs N153S and V154M.^40,41^ Similar to SAVI patients, these “SAVI” mice spontaneously develop a skin and lung disease and suffer premature death. Surprisingly, the pathology induced by constitutive STING activation seems to be independent of type I IFN because the skin and lung manifestations persisted when the mice were crossed with *Irf3*-deficient mice (for the N153S STING variant)^40^ and with *Ifnar*-deficient mice that were deficient in the IFN-α/β receptor (for the V154M STING variant).^41^ Interestingly, the N153S SAVI mouse model presented with T and NK cytopenia and impaired T cell proliferation.^40^ The V154M SAVI mouse model developed broad lymphocyte developmental defects involving T, B, and NK cells in addition to impaired T cell proliferation and hypogammaglobulinemia.^41^ Thus, the V154M mouse model experiences hyperactivation of STING that seems to cause a severe combined immunodeficiency (SCID)-like phenotype. In addition, the N153S SAVI mouse model has recently been shown to develop a combined innate and adaptive immunodeficiency that leads to pulmonary fibrosis upon viral infection.^42^ Although STING hyperactivation is difficult to study in humans, a recent report supports that at least the T cell imbalance is caused by impaired proliferation observed in V155M SAVI patient cells.^43^ Furthermore, STING activation affected T cell proliferation independent of TBK1, IRF3, and type I IFNs.

Modulation of the cGAS-STING pathway has attracted attention in recent years with the discovery of cGAS inhibitors^44,45^ in addition to several STING agonists that are used in cancer therapy.^46,47^ Despite the currently limited understanding of how STING hyperactivation leads to inflammation, it remains highly desirable to identify compounds that can directly target and inhibit STING signaling. Thus, STING signaling as an important contributor to inflammatory diseases motivated us^48^ and the laboratory of Andrea Ablasser^49^ to discover novel STING inhibitors. Interestingly, the independently described compounds are remarkably similar in reactivity and target the same STING residues to exert their inhibitory effects.

This mini-review describes recent advances in the understanding of STING signaling and the mechanistic background for the mechanisms by which these novel inhibitors block STING signaling.

## Sting activation and translocation from Er-to-Golgi

A full decade has passed since STING was first described as an important innate signaling molecule.^1,50,51^ STING was identified as a strong inducer of type I IFNs through the activation of TANK-binding kinase 1 (TBK1) and, subsequently, of the transcription factor IFN regulatory factor 3 (IRF3) and nuclear factor kappa B (NF-κB).^1,2,52,53^ Interestingly, STING was demonstrated to be important for resistance to infection by both RNA and DNA viruses. However, whereas STING was indispensable to the induction of IFNβ in response to cytosolic DNA, it was not involved in responses to cytosolic RNA, as shown by the direct delivery of RNA into the cytosol.^1,2^ Although activation of the cGAS-STING pathway has been implicated during infection by RNA viruses, such as with the Dengue virus,^54,55^ the importance of STING in the resistance to RNA viruses remains unclear.

In sharp contrast, the current understanding of the molecular mechanism that underlies the activation of STING downstream of cytosolic DNA sensing has progressed significantly. First, cyclic dinucleotides (CDNs) were identified as powerful STING-activating agents.^5^ Then, cGAMP (cyclic GMP-AMP) was identified as the mammalian CDN formed in response to cytosolic DNA and to infection by a DNA virus.^9,12^ These discoveries were followed by several independent reports that demonstrated that the enzyme cGAS (cyclic GMP-AMP synthase) was a cytosolic DNA sensor and was responsible for DNA-induced cGAMP production upstream of STING activation.^10,11,56^ The cytosolic DNA being sensed by cGAS can originate from various sources, including the nucleus^57,58^ and the mitochondria.^59^

In essence, although other sensor molecules have been demonstrated to be important for its function,^60^ cGAS is now recognized as the primary sensor of cytosolic DNA and thus as being indispensable for the downstream STING-dependent induction of type I IFNs. However, STING activation can also occur independently of cGAS sensing, i.e., during virus-cell fusion,^14^ direct sensing of bacterial CDNs^5^ or DNA damage.^61^

After CDN binding, STING translocates from the endoplasmic reticulum (ER) to perinuclear compartments including the Golgi body, endosomes, and autophagy-related compartments.^2,62^ Interestingly, blocking ER-to-Golgi membrane traffic with brefeldin A or ER-to-ER-Golgi-intermediate-compartment (ERGIC) with *Shigella* effector protein IpaJ, abolishes the STING-dependent signaling events that include the phosphorylation of TBK1 and the transcription factor IRF3 for the subsequent type I IFN induction.^2,63,64^ The requirement for translocation has recently gained additional support as the knockdown of Sar1, a small GTPase that regulates coat protein complex II (COP-II)-mediated ER-to-Golgi traffic, was demonstrated to inhibit the translocation of STING from the ER, as well as the binding of TBK1 to STING.^65^ It is therefore intriguing that some SAVI STING variants localize to the perinuclear compartments, including the Golgi body, even without DNA stimulation or STING-activating ligands.^63^ Likewise, the variants of the murine equivalent of SAVI C205Y, R280Q, and R283G (corresponding to the human SAVI variants C206Y, R281Q, and R284G),^37^ also localize to the perinuclear compartments and Golgi body.^65^ Consequently, the perinuclear localization of STING in the absence of STING ligands appears to be a common feature of all hyperactive SAVI variants.^63,65^ Furthermore, even for the hyperactivated SAVI variants, the binding of TBK1 was inhibited when STING was trapped in the ER.^65^ Together, these results imply that post-ER compartments contribute to the activation of STING.

The molecular mechanism underlying the translocation of STING from the ER has not been clarified. One possible mechanism could involve conformational changes induced in STING upon binding to CDNs, enabling the COP-II components to recognize the tentative export-signal sequences of STING. These changes may create binding sites for the COP-II components and/or release STING from a tethering protein that localizes it to the ER. Recently, the Ca^2+^ sensor STIM1 has been implicated in maintaining the resting state of the STING pathway by retaining STING at the ER membrane.^66^ It could be speculated that Ca^2+^ release from the ER upon virus-cell membrane fusion^14^ activates STING via STIM1. In summary, the translocation of STING seems to be essential for its activation.

## Palmitoylation is necessary for sting signaling

The translocation of proteins between cellular membranes is often accompanied by lipid-based posttranslational modifications.^67^ In particular, the presence of palmitate on a protein can affect how the protein interacts with lipids and other proteins in a membrane compartment.^68^ The ER-associated member of the palmitoyltransferase family, ZDHHC1, has previously been identified as a positive regulator of STING-dependent signaling,^69^ although its enzymatic activity was not required for STING activation. Along the same lines, it was more recently demonstrated that STING requires palmitoylation for its activation and subsequent type I IFN responses.^70^ Cysteine (Cys) residues in proteins are the main targets for the covalent attachment of the 16-carbon palmitic acid, and mammalian STINGs have several conserved Cys residues that are localized either in the cytoplasmic region or in the transmembrane region.^71^ Through the analysis of STING cysteine mutants, the sites of STING palmitoylation were identified as being cysteines 88 and 91. Furthermore, it was suggested that the protein palmitoyltransferases DHHC3, DHHC7, and DHHC15 contribute to the palmitoylation of Cys88/91 of STING in the Golgi body.^70^ Interestingly, the introduction of Cys88/91 mutations in the gain-of-function STING variants was sufficient to inhibit the activation of IRF3, IFNβ or NF-κB in reporter assays, indicating that the SAVI variants, as well as wild-type STING, require palmitoylation for their activity.^70^ Depalmitoylation is carried out by the acylprotein thioesteraser;^68^ nevertheless, the depalmitoylation of STING was not observed during its transport from the Golgi body to the degradation compartments.^70^ This highlights the potential for STING palmitoylation to be pharmaceutical target.

## Targeting sting palmitoylation involves cysteine alkylation

The central role of STING in the pathology of several inflammatory diseases has initiated an intense search for potential inhibitors of STING. Recently, our group identified nitro-fatty acids (NO_2_-FAs) as potent inhibitors of STING signaling.^48^ Simultaneously, the laboratory of Andrea Ablasser identified nitrofurans as small-molecule inhibitors of STING signaling.^49^ Remarkably, the NO_2_-FAs and the nitrofuran compounds seem to inhibit STING through closely related mechanisms involving cysteine alkylation.

**Fig. 1:**
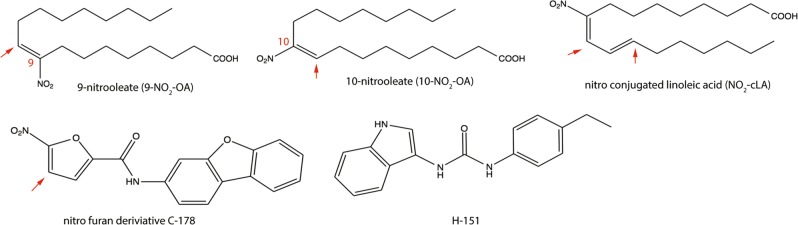
NO_2_-FAs and nitrofuran derivatives that inhibit STING signaling. The red arrows indicate the sites that can participate in a Michaels addition reaction with STING cysteines

**Fig. 2:**
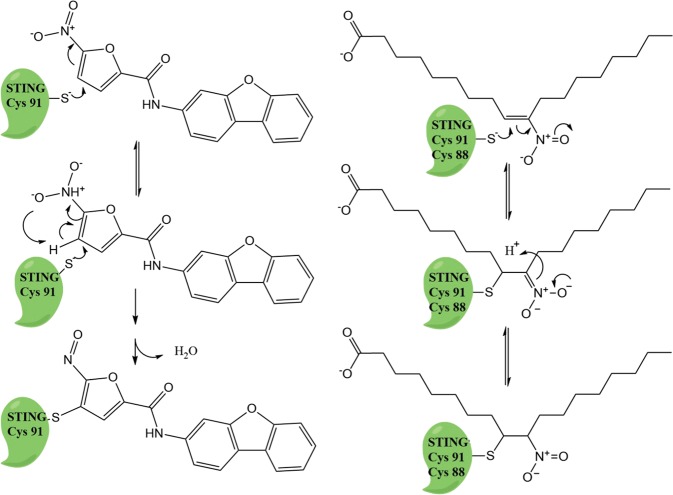
Proposed chemical reaction between the electrophilic nitrofuran (left), nitroalkene-containing inhibitors (right) and STING Cys 88 and 91

The reactivity of both types of compounds is centered around reactive nitro-groups **(**Fig. 1**)**. The strong electron withdrawing properties of the nitro-groups in the nitroalkenes and nitrofurans make them excellent Michael acceptors and reactive with thiols such as those present in cysteines. It is important to consider that the concentration of the main intracellular thiol-containing biomolecule is glutathione (2–17 mM) and that the total reduced protein thiol content is ~10–50 mM.^72,73^ As both series of inhibitors directly react with cysteine 91 and NO_2_-FA reacts with cysteine 88, the mechanism by which STING becomes targeted under these highly reducing and thiol-abundant conditions remains unknown. In this regard, the thiol *p*K_a_ is a strong determinant of the target cysteine reactivity toward electrophiles, and it is modulated by structural determinants. Basic neighboring amino acids can stabilize the ionized state of the cysteine, which is the reactive state, greatly increasing its reactivity by lowering its *p*K_a_. In addition to determining the *p*K_a_, protein pocket structural properties establish the kinetics of inhibitor docking, adduct formation, and reversibility; the latter is only observed in the case of NO_2_-FA. The structural influence and the relevance of the electrophilic reactivity on the inhibition of STING is highlighted by the selectivity displayed by the nitrofuran compound C-176 when compared to that of other reactive nitrofuran molecules that have different sidechain substituents or related unreactive structures^49^ **(**Fig. 2**)**. One of the particular characteristics of nitrofuran is that the initial Michael adduct formed undergoes a rearrangement followed by dehydration to yield an irreversible adduct. This is in strong contrast with NO_2_-FAs, the other group of selective inhibitors, which undergo reversible Michael additions with cysteines. One exception of the strong reactivity is exemplified by compound H-151.^49^ Using click chemistry approaches and whole protein mass spectrometry, the authors have shown that this compound covalently interacts with STING, despite containing functional groups that would indicate a lower reactivity with thiols when compared to nitroalkenes and nitrofurans.

NO_2_-FAs are a recently discovered group of bioactive lipids with anti-inflammatory and tissue protective functions.^74^ The NO_2_-FAs are constantly formed in the gastrointestinal tract during digestion, and they are absorbed into and distributed through the systemic circulation.^75,76^ In addition, they can be formed locally at sites of inflammation and during ischemia-reperfusion events. Their levels were found to be increased in the peritoneum during lipopolysaccharide-induced sepsis^77,78^ and in heart tissue during ischemic conditions in rodents.^79^ More recently, we reported the formation of NO_2_-FAs in response to HSV-2 infection in addition to their role as novel inhibitors of STING signaling.^48^ Fatty acids containing conjugated double bonds are the main targets of nitration, showing several orders of magnitude higher yields of formation than monounsaturated or bis-allylic polyunsaturated fatty acids.^80^ Thus, a limited number of endogenous NO_2_-FAs have been identified, and this is closely related to the availability of conjugated fatty acids these reactions use as substrates.

In general, the NO_2_-FAs posttranslationally modify their target molecules through Michael addition reactions, resulting in *S*-nitro-alkylation.^74,81^ In the case of STING, the NO_2_-FAs nitro-alkylate the thiol groups of cysteines at positions 88 and 91 in STING.^48^ Interestingly, the cysteine at position 91 is also targeted by the nitrofuran molecules.^49^ Both cysteines are located in the N-terminal region of STING in close proximity to the proposed transmembrane domains. The location of Cys88 and Cys91 may favor a specific contact with the electrophilic NO_2_-FAs.

**Fig. 3:**
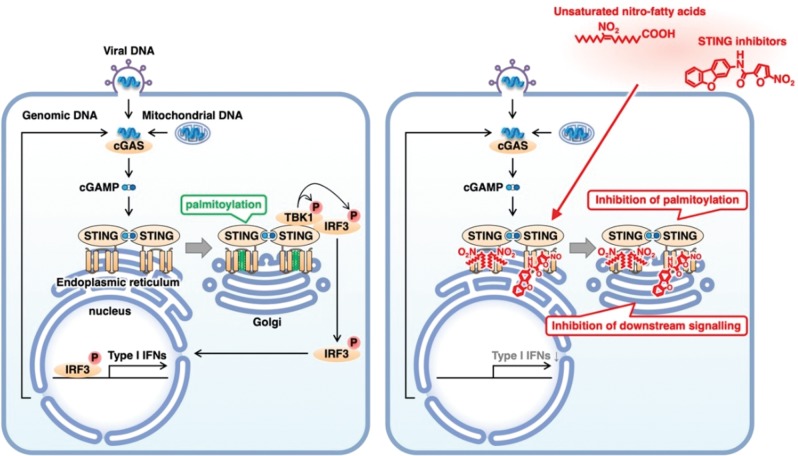
Alkylation of STING cysteines 88/91 inhibits STING palmitoylation and signaling

As described above, STING Cys88/91 are targets of palmitoylation, which is a modification that is required for STING activation and important for STING clustering in the trans-Golgi body network.^70^ Palmitoylation also seems to be important for the recruitment and phosphorylation of TBK1 and, subsequently, the phosphorylation of IRF3.^70^ Indeed, the treatment of cells with NO_2_-FAs or nitrofuran molecules abolishes STING palmitoylation and, subsequently, the phosphorylation of TBK1 and IRF3 in response to stimulation with STING agonists.^48,49^ Thus, targeting STING palmitoylation with NO_2_-FAs or nitrofuran molecules inhibits STING signaling **(**Fig. 3**)**.

Gain-of-function mutations in STING drive devastating inflammatory diseases, such as SAVI, and it was therefore reasonable to test whether NO_2_-FA-induced inhibition of STING could override the genome-encoded hyperactivity of STING in patient-derived cells. Intriguingly, NO_2_-FA treatment of immortalized SAVI patient-derived fibroblasts inhibits STING signaling by abolishing TBK1 phosphorylation and ultimately suppresses the release of type I IFNs.^48^ Furthermore, treatment of *Trex1*-deficient mice with the nitrofuran compounds reduced the serum levels of type I IFNs and impacted inflammatory markers in the heart.^49^ Together, these two independent studies come to the following conclusion: STING palmitoylation is a valid pharmacological target for the inhibition of STING signaling and thus for the treatment of STING-dependent pathologies.

## Summary and conclusions

A growing number of reports place STING as a central driver of pathology in a series of autoinflammatory and autoimmune disorders, such as SAVI, SLE, and AGS. In addition, STING has recently been reported to play a role in neuro-inflammation.^82^ The contribution of STING is either direct or indirect, depending on the type of disease. Mutations in genes important for diminishing cytosolic DNA loads, such as *TREX1,* play an indirect role in disease because rising levels of cytosolic DNA can activate STING via cGAS. However, gain-of-function mutations in the STING-encoding gene are the direct driving force establishing the pathological condition in SAVI. Collectively, the use of inhibitors of STING signaling may present a new and effective treatment strategy for inflammatory diseases. Interestingly, the efficacy of STING inhibitors in such conditions will not depend on which downstream effectors are causing the symptoms. The NO_2_-FAs and nitrofuran compounds seem to broadly inhibit STING signaling; hence, they potentially suppress the broad range of symptoms experienced by the patients. Five Phase I clinical trials successfully concluded that NO_2_-FAs are well tolerated. Furthermore, the lead compound, 10-nitro oleic acid (CXA-10), is currently being assessed in two phase II clinical trials for the treatment of focal segmental glomerulosclerosis (clinicaltrials.gov: NCT03422510) and pulmonary arterial hypertension (clinicaltrials.gov: NCT03449524). To date, most of the anti-inflammatory activities of NO_2_-FA have been attributed to the inhibition of NF-κB signaling.^83,84^ However, the novel targeting of STING by NO_2_-FAs suggests a new mechanism of inhibition. Thus, these anti-inflammatory lipids represent a novel strategy for the treatment of patients with STING-derived inflammatory conditions.
